# LIF-Free Embryonic Stem Cell Culture in Simulated Microgravity

**DOI:** 10.1371/journal.pone.0006343

**Published:** 2009-07-23

**Authors:** Yumi Kawahara, Tomotaka Manabe, Masaya Matsumoto, Teruyuki Kajiume, Masayasu Matsumoto, Louis Yuge

**Affiliations:** 1 Department of Clinical Neuroscience and Therapeutics, Graduate School of Biomedical Sciences, Hiroshima University, Minami-ku, Hiroshima, Japan; 2 Space-Bio Laboratories Y. K., Hiroshima University, Minami-ku, Hiroshima, Japan; 3 Division of Bio-Environmental Adaptation Sciences, Graduate School of Health Sciences, Hiroshima University, Minami-ku, Hiroshima, Japan; 4 Department of Pediatrics, Graduate School of Biomedical Sciences, Hiroshima University, Minami-ku, Hiroshima, Japan; Baylor College of Medicine, United States of America

## Abstract

**Background:**

Leukemia inhibitory factor (LIF) is an indispensable factor for maintaining mouse embryonic stem (ES) cell pluripotency. A feeder layer and serum are also needed to maintain an undifferentiated state, however, such animal derived materials need to be eliminated for clinical applications. Therefore, a more reliable ES cell culture technique is required.

**Methodology/Principal Findings:**

We cultured mouse ES cells in simulated microgravity using a 3D-clinostat. We used feeder-free and serum-free media without LIF.

**Conclusions/Significance:**

Here we show that simulated microgravity allows novel LIF-free and animal derived material-free culture methods for mouse ES cells.

## Introduction

ES cells readily proliferate and differentiate but also undergo unexpected spontaneous differentiation. Mouse ES cells are typically maintained on a feeder layer of mouse embryonic fibroblasts (MEFs). Recently, MEF-conditioned media was shown to support the proliferation of mouse ES cells, eliminating the need of a feeder layer. It was subsequently demonstrated that MEFs inhibit ES cell differentiation by producing the IL-6 family cytokine, LIF [1]. Adding recombinant LIF to mouse ES cell culture medium removes the need for MEF.

We are currently culturing cells in a microgravity environment to determine their cellular responses to physical stimulation. Previous studies have shown that microgravity suppresses the differentiation of human osteoblast cells [2], human hematopoietic progenitor cells [3], and rat myoblasts [4]. Yuge *et al.* [5] showed that human mesenchymal stem cells cultured under simulated microgravity maintained their undifferentiated state. When these cells were transplanted into cartilage defective mice, they differentiated into hyaline cartilage and also had a high survival rate. Indeed, some studies have indicated that microgravity suppresses cellular differentiation thereby providing a potentially effective means to maintain stem cell characteristics.

We found that mouse ES cells could be maintained in feeder-free and serum-free culture conditions without LIF in simulated microgravity using the 3D-clinostat.

## Results

**Figure 1:**
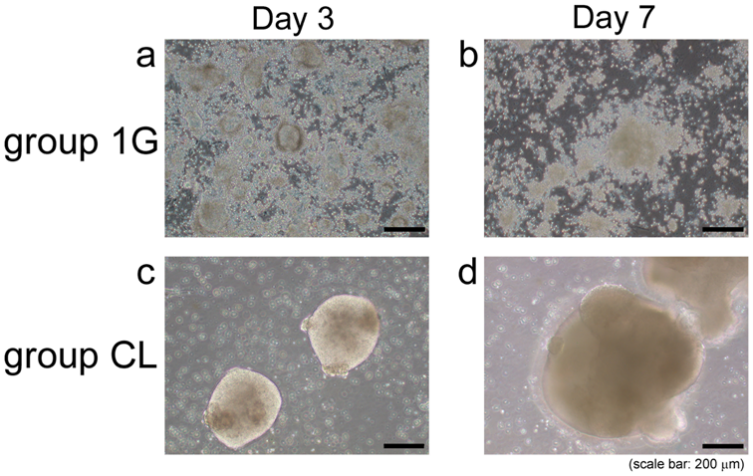
Morphological changes of cultured mouse ES cells on Day 3 and 7. All the cells became oval cell shapes and flattened, the phenotype of differentiated ES cells in group 1G (a, b). The cells of group CL showed the formation of cell spheres (c, d).

**Figure 2:**
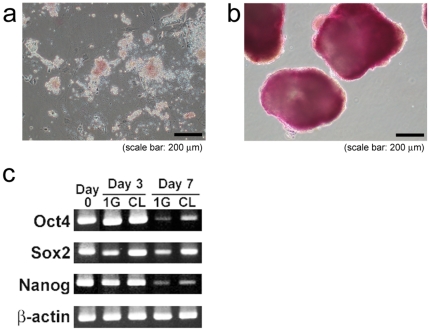
ALP staining of group 1G (a) and group CL (b), on Day 7. The cell spheres of group CL were positive for ALP. The cells of group CL expressed undifferentiated cell markers (c).

**Figure 3:**
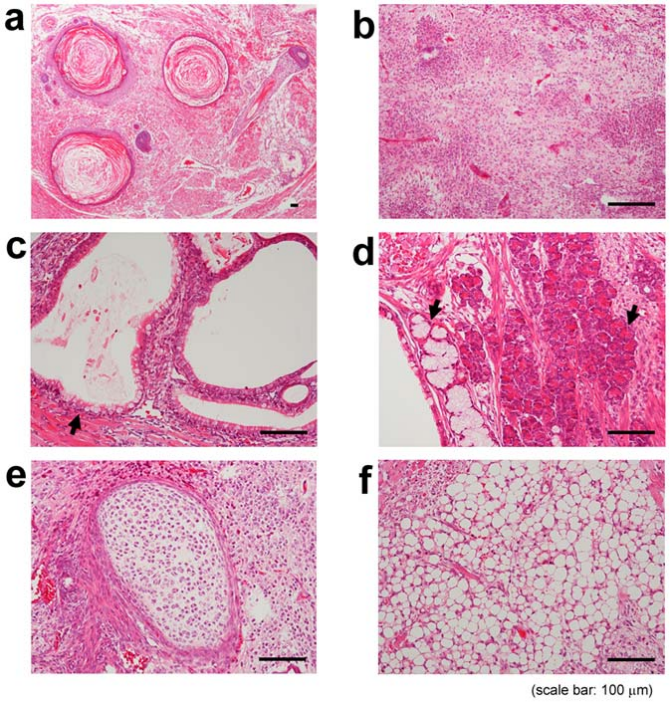
H & E staining of a teratoma on Day 35 following injection. The teratoma was generated by subcutaneously injecting cells grown in simulated microgravity for seven days. The teratoma contains a mixture of mature and immature elements derived from the ectoderm, endoderm and mesoderm in a haphazard distribution. Ectodermal derivatives include hair follicles (a) and glial tissue (b). Endodermal elements are glandular tissue (c), with a partial acinar structure (d). Mesodermal components are cartilage (e) and adipose tissue (f).

Group 1G cells (ES cells in normal 1G environment) were morphologically different from general ES cells. They included forms similar to those of differentiated cells (Fig. 1a,b). On the other hand, group CL cells (ES cells in simulated microgravity environment) formed many small spheres after three days of culture (Fig. 1c). These floating spheres grew bigger during the culture period (Fig. 1d). After seven days of culture, the number of cells in group CL was about eight-times the number for group 1G. We tested these spheres to determine if they were indeed ES cells, and not embryonic body cells. Alkaline phosphatase (ALP) staining results indicated that the mouse ES cells had grown in group CL without the usual support of LIF or the serum and feeder layer and were maintained in an undifferentiated state (Fig. 2a,b). After three days of culture, *Sox2* expression decreased in the cells of group 1G and *Oct-4* and *Nanog* expressions decreased by Day 7 (Fig.2c). Sphere-forming cells in group CL, however, maintained the expression of these undifferentiated markers. Moreover, we confirmed the pluripotency of cells cultured under simulated microgravity for seven days. Teratomas were only generated by subcutaneously injecting group CL cells into C57BL/6 mice. The teratoma contains a mixture of mature and immature elements derived from the ectoderm, endoderm and mesoderm in a haphazard distribution (Fig. 3).

## Discussion

Prolonged exposure of humans and animals to microgravity results in several well characterized physiological changes, including space flight–induced bone loss, anemia and immunosuppression. Data from astronaut studies and model microgravity experiments show that gravity regulates cell proliferation, differentiation, and function.

Microgravity conditions can be produced by space flight or by a free fall. However, the duration of microgravity conditions produced by a free fall is usually too short to alter cell growth and differentiation. Because of limited access to space flight, many efforts have been made to establish alternative methods for simulating microgravity on Earth, and a clinostat is considered to be a device for simulating microgravity. The 3D-clinostat is a recently developed device for generating multi-directional G force, resulting in an environment with an average of 10^−3^ G [2], [6]. Our previous studies demonstrated that cells cultured in a 3D-clinostat show suppression of cell differentiation [2], [4], [5].

Recently, animal-free techniques, such as feeder-free and serum-free methods, have been developed for ES cell culture. However, most methods have required LIF and culture vessels coated with animal derived materials, such as collagen, gelatin and complex matrix [7], [8]. We successfully developed a novel LIF-free simulated microgravity culture technique for mouse ES cells. In addition, our method does not require a feeder layer, serum, coating materials or trypsin to maintain the cells. Our results suggest that simulated microgravity provides a straightforward and effective means for stem cell culture. Moreover, our results corroborate previous reports [9] suggesting that *Sox2* plays an important role during ES cell proliferation. We anticipate that this culture technique will be just as effective for human ES cell culture. Further study is needed to determine how stem cells remain undifferentiated in a simulated microgravity environment.

## Materials and Methods

### 3D-clinostat

The 3D-clinostat, a multidirectional G force generator, was produced by MITSUBISHI HEAVY INDUSTRIES, LTD. (Kobe, Japan). By employing a simultaneous rotation on two axes, the 3D-clinostat cancels the cumulative gravity vector at the center around the device, producing an environment with an average of 10^−3^G over time. This is accomplished by rotating a chamber at the center of the device to disperse the gravity vector uniformly within a spherical volume, at a constant angular velocity (Undifferentiated pluripotent stem cell proliferation/differentiation regulation method and system, Japanese patent, publication number P2001-197182A, date of filing June. 28, 2001, and overseas patents, WO2004/061092 A1 PCT, 2004).

### Mouse ES Cells Culture

We purchased mouse ES cells (BRC6, derived from C57BL/6 mice) from the RIKEN BRC CELL BANK (Tsukuba, Japan), and cultured them with mouse embryonic fibroblasts according to a standard protocol. Then, 1.0×10^6^ mouse ES cells were seeded on OptiCell™ (Thermo Fisher Scientific Nunc brand, Rochester, NY), and cultured in normal 1G conditions (group 1G) or 10^−3^ G conditions (group CL), using the 3D-clinostat (Day 0). We used feeder-free and serum-free ESF-C media (Cell Science and Technology Institute Inc., Sendai, Japan) without LIF. The culture vessels were not coated. The culture medium was not changed for seven days.

### ALP Staining

Cells were examined for ALP using an Alkaline Phosphatase Detection Kit (Millipore Co., Billerica, MA) according to the manufacturer's supplied protocol. The stained cells were examined using an inverted phase-contrast microscope (Eclipse TE 300; Nikon Co., Tokyo, Japan).

### RT-PCR

Samples were collected using ISOGEN (NIPPON GENE Co., Ltd., Toyama, Japan), and RNA was isolated according to the supplied protocol. Reverse transcription was performed with ReverTra Ace -α- (Toyobo Co. Ltd., Osaka, Japan). Using cDNA as the template, PCR was performed using BD Advantage™ 2 PCR Kits (BD Biosciences Clontech, Palo Alto, CA). We used *Oct-4*, *Sox2*, and *Nanog* as the pluripotency markers and *β-actin* as a housekeeping gene. The sequence of the prepared primers as well as PCR conditions is shown in Table 1.

**Table 1:** Primers used for PCR and PCR conditions.

target gene (product size)	primer sequences (sense and antisense)	PCR condition	cycles
*Oct-4* (430 bp)	5′-CCG TGA AGT TGG AGA AGG TG -3′		30 cycles
	5′-TGA TTG GCG ATG TGA GTG AT -3′		
*Sox2* (467 bp)	5′-AGA ACC CCA AGA TGC ACA AC -3′	95°C, 30 s	33 cycles
	5′-CAT GTA GGT CTG CGA GCT GG -3′	58°C, 30 s	
*Nanog* (400 bp)	5′-GCA GAT GCA AGA ACC TCC TC -3′	68°C, 60 s	30 cycles
	5′-CGT AAG GCT GCA GAA AGT CC -3′		
*β-actin* (450 bp)	5′-GAG AGG GAA ATG GTG CGT GA -3′		28 cycles
	5′-ACA TCT GCT GGA AGG TGG AC -3′		

### Determination of Cell Differentiation

At Day 7 of culture, the cells of group 1G and group CL were assessed for their ability to differentiate. We subcutaneously injected 3.0×10^6^ cells in 200 µl of PBS into a C57BL/6 mouse. After 35 days, the mice were killed and teratomas were removed, and fixed overnight in 10% formalin. Paraffin sections were used for hematoxylin and eosin (H & E) staining, and these were examined using a multifunctional microscope (BZ-9000; KEYENCE Co., Osaka, Japan).
